# Salvage En Bloc Resection for Refractory Recurrent Pure Testicular Seminoma: A Case Report

**DOI:** 10.1002/ccr3.70206

**Published:** 2025-02-13

**Authors:** Mohammad Mohammadianpanah, Susan Andalibi, Mansour Ansari, Hamid Nasrollahi, Ahmad Mosalaei, Shapour Omidvari, Maral Mokhtari, Niloofar Ahmadloo, Ehsan Mohammad Hosseini

**Affiliations:** ^1^ Department of Radiation Oncology, Namazi Hospital Shiraz University of Medical Sciences Shiraz Iran; ^2^ Department of Pathology, School of Medicine Shiraz University of Medical Sciences Shiraz Iran; ^3^ Department of Neurosurgery, Namazi Hospital Shiraz University of Medical Sciences Shiraz Iran

**Keywords:** chemotherapy, seminoma, surgical resection, testicular malignancy

## Abstract

Treating recurrent or cisplatin‐refractory germ cell tumors remains a clinically problematic situation. Although testicular cancer has a good overall cure rate, some patients with metastatic GCTs who did not respond to initial treatments have few therapeutic alternatives and face significant fatality rates. This case showed the successful implementation of radical surgery for patients with refractory seminoma in clinical practice.

## 1 Introduction

Testicular malignancies are the most prevalent malignant tumors in males aged 15–35 years [1]. Around 90% of testicular malignancies are germ cell tumors (GCTs), which can be categorized as seminoma or nonseminomatous germ cell tumors (NSGCTs). GCTs have high sensitivity to chemotherapy, with conventional platinum‐based treatment resulting in a cure rate of up to 80% for individuals with metastatic illness. Patients who have relapsed or are resistant to cisplatin have a very low chance of survival, with less than 5% achieving long‐term survival. At present, there is no documented treatment for people who experience future relapse [2, 3]. Although advancements have been made in treating individuals with advanced GCTs, 10%–50% of those with metastatic illness will relapse during disease progression. Salvage treatment is more intricate and less confirmed than initial treatment due to its infrequency, diverse patient groups, and the significant influence of prognostic variables.

We report a case of seminoma that did not respond to two rounds of chemotherapy and radiotherapy but was successfully treated with surgery.

## 2 Case History

**FIGURE 1:**
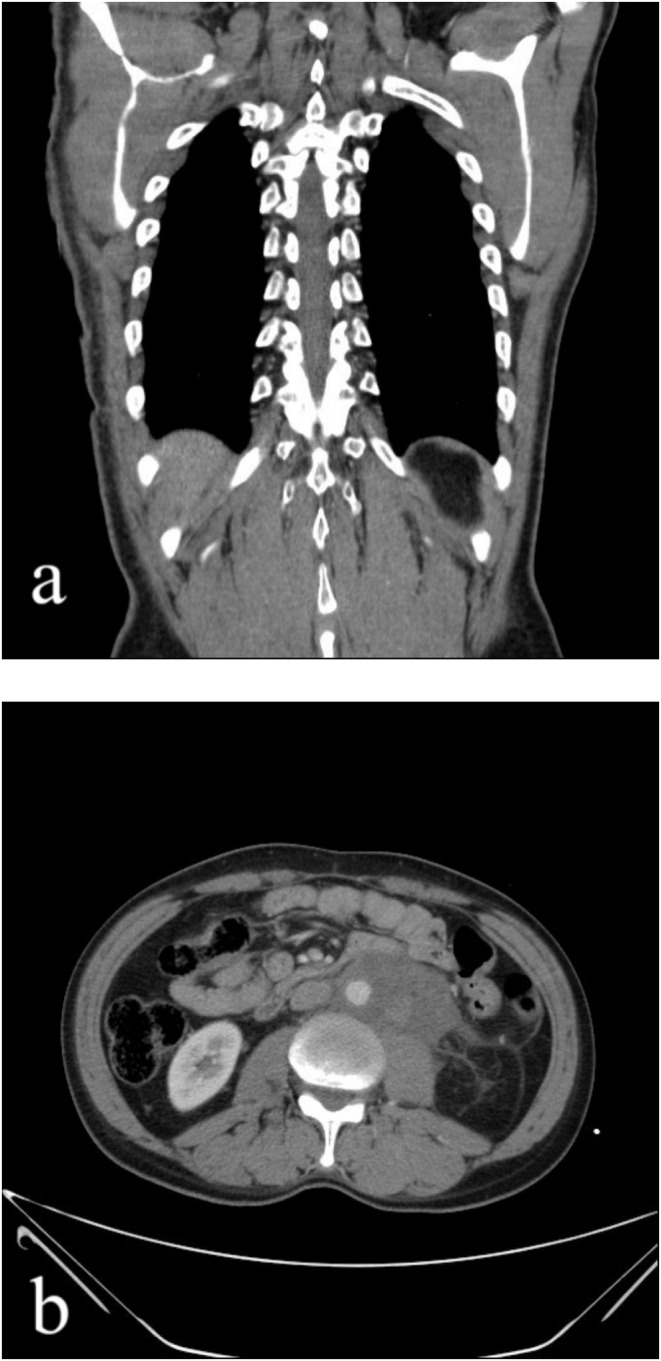
Contrast‐enhanced computed tomography (CT) in coronal view (a) and axial view (b) showing a 14 × 12 × 11 cm soft tissue mass in the left retroperitoneum suggestive of a metastatic tumoral mass.

In August 2016, a 44‐year‐old male with no previous medical or family history arrived with a tumor in his left testicle. A testicular ultrasound revealed a sizable tumor measuring approximately 5 × 4 × 3/5 cm in his left testis. He had a radical surgical removal of the testicle through the inguinal area. The pathology report indicated the presence of classic seminoma with necrosis and vascular invasion, although the tumor was not seen in the epididymis or spermatic cord margins. After the surgery, the patient opted for a watch‐and‐wait approach and did not undergo any additional treatment. In May 2018, around 18 months later, the patient showed signs of a growing mass and fullness in the flank area. An abdominal ultrasonography revealed a sizable lobulated mass measuring 12.3 × 11.3 cm in the left retroperitoneal region, leading to left‐sided hydronephrosis. A 14 × 12 × 11 cm soft tissue mass was identified in the left retroperitoneal area on contrast‐enhanced computed tomography (CT), indicating a metastatic tumoral mass (Figure 1).

The levels of alpha‐fetoprotein (AFP) and beta‐human chorionic gonadotropin (B‐HCG) were within the normal range at 2.1 ng/mL and 4.6 mIU/mL, respectively.

**FIGURE 2:**
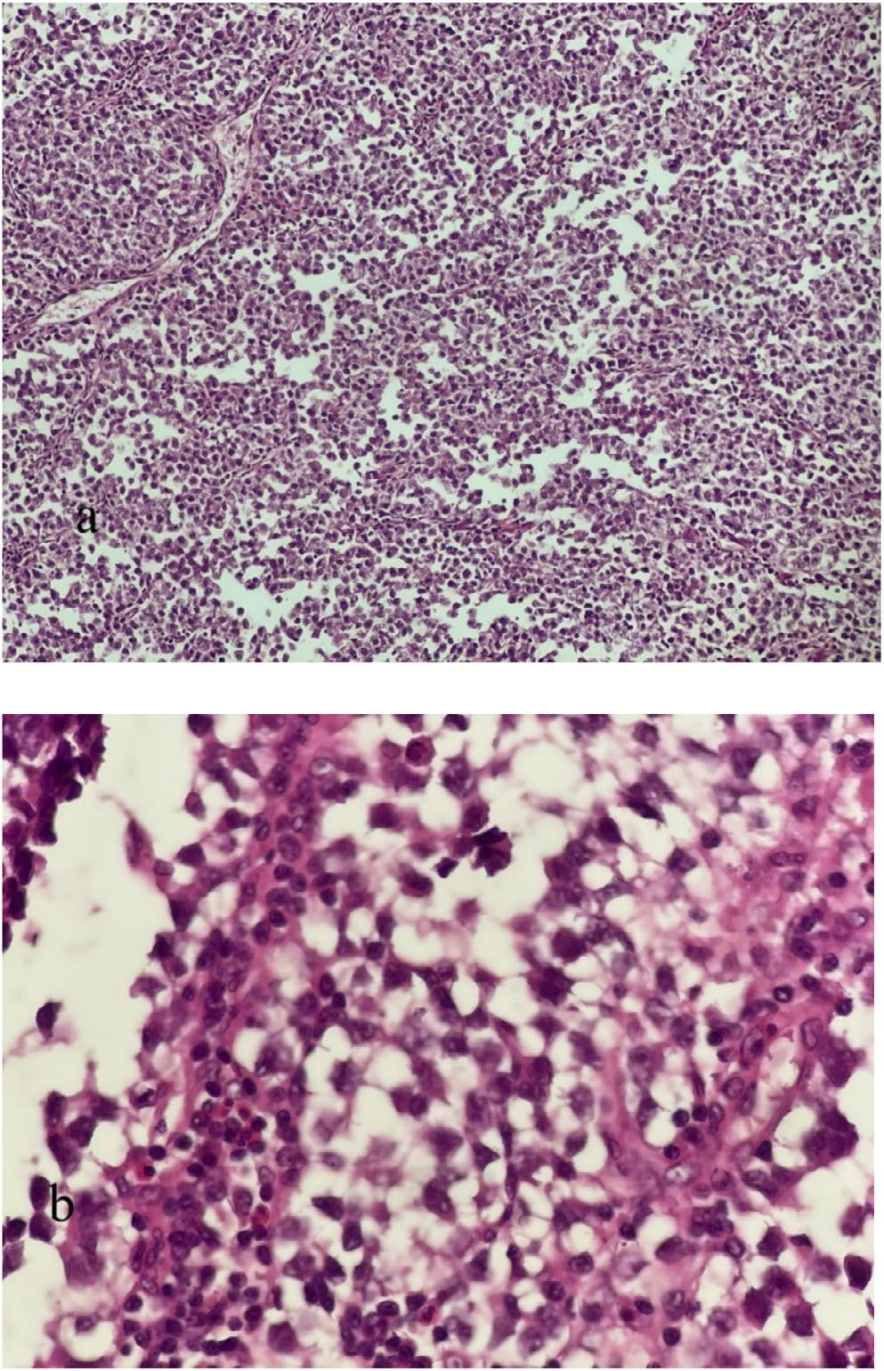
Cell sheets with marked nuclear atypia in a lymphocytic background, H&E. (a) ×100. (b) ×400.

**FIGURE 3:**
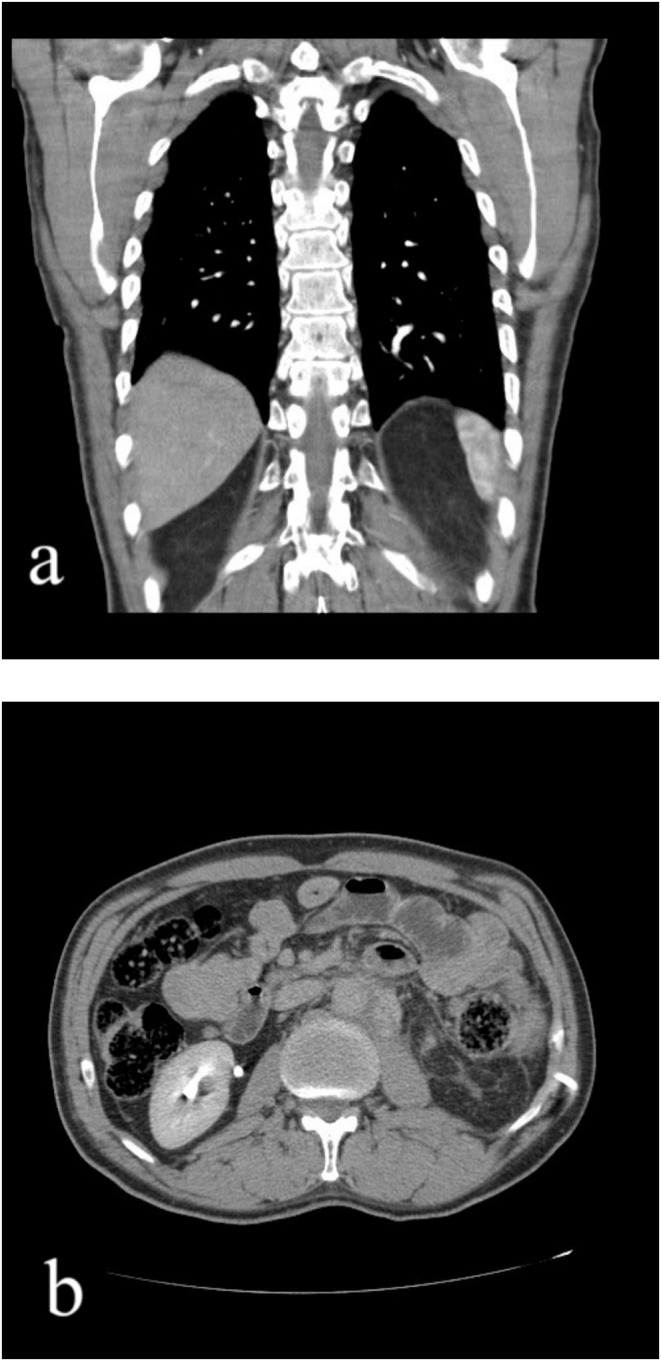
A chest‐abdominal and pelvis CT in coronal view (a) and axial view (b) showing a residual mass of about 11 × 7 × 4 cm with a partial response to radiotherapy, without distant metastasis.

A tru‐cut biopsy was conducted on a large paraaortic tumor. The pathology study showed metastatic classic seminoma, which was confirmed by immunohistochemistry (IHC). The patient had four cycles of bleomycin + etoposide + cisplatin (BEP) therapy. Following imaging tests including ultrasonography and CT scan, which indicated no improvement with the initial chemotherapy, the patient underwent four rounds of second‐line chemotherapy with the VIP regimen (vinblastine, ifosfamide with mesna protection, and cisplatin). Despite undergoing two rounds of chemotherapy, a recent ultrasonography and CT scan revealed a persistent paraaortic mass (matted lymph nodes) in the upper abdomen measuring 15 × 14 × 10 cm, with no notable improvement in response to the treatment. Therefore, the patient was referred to the Radiation Oncology department due to an unresectable metastatic paraaortic tumor. During the physical examination, a palpable fullness was noted on the left flank, which was not associated with pain or tenderness. AFP and B‐hCG tumor markers were within normal limits. A nephrostomy tube was inserted to treat left‐sided hydronephrosis. Furthermore, a tru‐cut needle biopsy of the paraaortic tumor was performed to eliminate other potential diseases including teratoma. Metastatic classic seminoma was verified through pathologic investigation and IHC (Figure 2). Based on a comprehensive review of all images and pathological data, the patient has a solitary para‐aortic mass that cannot be surgically removed and does not have distant metastases. Hence, the individual received external beam radiation in two stages. Initially, a 20‐Gy dose was administered to target the retroperitoneal bulk and para‐aortic lymph nodes. During the second phase, a further dose of 16 Gy was administered to the large retroperitoneal bulk. Two months post‐radiotherapy, a CT scan of the chest, abdomen, and pelvis revealed a remaining mass measuring around 11 × 7 × 4 cm, showing some improvement after the radiotherapy, with no signs of distant spread (Figure 3). The patient was sent to the surgeon for major surgery due to inadequate response to chemotherapy and radiotherapy.

In June 2018, a comprehensive surgical procedure was carried out involving the removal of the left kidney and associated organs like the left colon, omentum, and lymph nodes. Following the detection of the remaining tumor in the tissue sample, the patient underwent four cycles of combination treatment with paclitaxel and carboplatin. Subsequently, all examinations, including tumor markers and scans of the chest, abdomen, and pelvis, showed no abnormalities over the 5‐year follow‐up period.

## 3 Differential Diagnosis

The differential diagnosis for testicular cancer includes conditions such as epididymal‐orchitis, testicular torsion, trauma, hematoma, leukemia, metastatic disease from other cancers (like lung or prostate cancer), testicular tuberculosis, and syphilitic gumma.

## 4 Conclusion and Result

Testicular tumors are the predominant malignant neoplasm in males aged 15–35 years; however, they can develop at any age or genetic background. Caucasians have a higher prevalence of this condition than Africans and Asians. They are more common in the right testis, with a frequency of 52.3%. Around 90% of testicular malignancies are GCTs found in both gonadal and extragonadal GCT locations. GCTs can be categorized as either seminoma or NSGCTs. Testicular seminoma, while being a cancerous tumor, has a high cure rate of approximately 95% when detected early. Around 15% of pure seminoma cases are diagnosed with regional or distant metastases at the time of diagnosis. In cases of seminoma, lactate dehydrogenase (LDH) levels may be raised, and occasionally there may be a modest increase in B‐hCG, but AFP is never secreted. Testicular tumors typically manifest as a painless enlargement of the testis that is discovered by the patient, their spouse, or during a medical examination. Testicular tumors are typically identified by a solid mass in the testicle that has a firm consistency. Testicular tumors typically spread through the bloodstream and lymphatic system [4]. Retroperitoneal metastases and gastrointestinal involvement are infrequent occurrences. Chest, abdominal, and pelvic CT scans, together with brain MRI, are utilized for illness staging. Patients are categorized into three distinct groups based on tumor markers and metastases to determine prognosis. Treatment is based on these categories [5]. Radical orchiectomy is the typical procedure for primary tumors. Histopathology of the specimen confirms the diagnosis and distinguishes the kind of GCT [6]. Patients with stage 1 seminoma have post‐orchiectomy choices such as active surveillance, retroperitoneal lymph node dissection, or chemotherapy (1 or 2 cycles). Chemotherapy is the primary treatment for most advanced cancers. Although advancements have been made in treating individuals with advanced GCTs, 10%–15% of those with metastatic disease will still relapse during the course of the illness. Managing relapse following cisplatin‐based chemotherapy and probable residual mass removal is more complex due to its rarity, diverse patient groups, and significant influence of prognostic variables. Around 50% of patients who experience their first relapse after BEP treatment can be effectively treated. Before starting any salvage treatment, it is important to confirm the failure of the initial treatment, identify any metastatic locations and determine the extent of the disease, evaluate established prognostic markers, and select the most effective salvage technique. Unlike initial therapy, treatment options are less clearly defined in the salvage situation following relapse.

Three relapse scenarios occur following the completion of the first treatment.

(1) Early relapse is characterized by relapses occurring within 2 years after finishing the original therapy and is the most frequent type.

Two less prevalent conditions are as follows: (2) Platinum‐refractory disease; disease progression occurring directly beneath or within 4 weeks after completion of initial cisplatin‐based therapy.

(3) Late relapse can occur more than 2 years after the initial therapy, affecting 1%–3% of all patients treated for severe illness.

Patients receive either standard salvage cisplatin‐based chemotherapy or high‐dose chemotherapy based on prognostic variables and selective patient criteria.

Conventional‐dose chemotherapy (CDCT) can effectively save approximately 15%–70% of patients during their initial salvage effort. Long‐term remissions in salvage therapy are significantly less common compared to initial treatment. Platinum‐based chemotherapy is the primary treatment for salvage chemotherapy in relapse cases. While there is no established standard strategy, VIP or TIP (cisplatin, paclitaxel, and ifosfamide) for 4 cycles are frequently utilized. Some clinicians prescribe a combination of paclitaxel, ifosfamide, and cisplatin as second‐line therapy for patients with relapsed testicular GCTs [7, 8, 9]. This chemotherapy regimen is currently the most commonly utilized for relapsed cases and has demonstrated compelling effectiveness. Another treatment for relapsed GCTs is high‐dose chemotherapy followed by stem cell rescue, which demonstrates no disparity in overall survival and progression‐free survival after 2 years when compared to platinum‐based chemotherapy [10]. HDCT is advised even for individuals who have not responded to many prior conventional therapies.

Salvage surgery is more successful than salvage chemotherapy in two key subgroups of patients: (1) patients with “growing teratoma” syndrome who show radiologic progression during or after initial chemotherapy but have normalized tumor markers; (2) patients with resectable late‐relapse GCTs that develop more than 2 years after cisplatin‐based initial chemotherapy. If possible, any remaining lesions that remain after chemotherapy after a relapse should be surgically removed. Approximately 20% of patients achieve effective surgical salvage, with a long‐term disease‐free survival rate ranging from 21% to 25%.

Országhová et al. mentioned that cisplatin resistance in GCTs has different cellular mechanisms. The mechanisms of cisplatin resistance, in general, are pre‐target, on‐target, and post‐target. Pre‐target resistance includes alterations occurring before cisplatin binds to DNA in the cell, on‐target resistance refers to alterations directly related to DNA‐cisplatin adducts, and post‐target resistance involves alterations in downstream signaling pathways of cisplatin‐mediated DNA damage that lead to apoptosis [11].

Debate surrounds the best systemic treatment for instances that cannot be operated on.

## 5 Discussion

Treating recurrent or cisplatin‐refractory GCTs remains a clinically problematic situation. Although testicular cancer has a good overall cure rate, some patients with metastatic GCTs who did not respond to initial treatments have few therapeutic alternatives and face significant fatality rates. Treatment options include salvage surgery in specific cases and multimodal treatment, such as HDCT, radiotherapy, and resection of remaining lesions based on their disease characteristics, response to previous treatment, and tumor size at the time of salvage therapy. Therapeutic options are generally based on adverse prognostic variables, resource availability, and patient and physician preferences due to the complexity of the problem, and lack of large randomized studies.

Refractory pure testicular seminoma in patients with recurrent stage I is rare. We present a case successfully treated with en bloc resection of a large paraaortic mass after two lines of failed salvage chemotherapy and radiotherapy, highlighting the potential of surgical intervention in challenging cases.

The successful implication of this case suggests a potential role of radical surgery for patients with refractory seminoma in clinical practice.

## Author Contributions

**Mohammad Mohammadianpanah:** conceptualization, investigation, supervision, validation, writing – review and editing. **Susan Andalibi:** methodology, supervision, visualization, writing – original draft. **Mansour Ansari:** writing – review and editing. **Hamid Nasrollahi:** methodology, writing – review and editing. **Ahmad Mosalaei:** investigation, methodology, writing – review and editing. **Shapour Omidvari:** investigation, methodology, writing – review and editing. **Maral Mokhtari:** writing – review and editing. **Niloofar Ahmadloo:** writing – review and editing. **Ehsan Mohammad Hosseini:** methodology, writing – review and editing.

## Ethics Statement

All procedures performed were under the institutional and/or the national research committee's ethical standards and the 1964 Helsinki Declaration and later amendments or comparable ethical standards. Shiraz University neurosurgery department board members supervised and approved this research on behalf of the Ethical Committee of Shiraz University of Medical Sciences.

## Consent

Written informed consent was obtained from the patient to publish this report. All authors have agreed to the submission and possible publication in the corresponding journal.

## Conflicts of Interest

The authors declare no conflicts of interest.

## Data Availability

The data supporting this study's findings are available on request from the corresponding author. However, due to privacy or ethical restrictions, the data are not publicly available.
